# Description of the Plate to Mr. Earle's Case of Suppuration of the Brain, Discovered by Dissection, but Not Suspected during Life

**Published:** 1810-03

**Authors:** 


*Description of the Plate to Mr.* EARLE'S *Case of Suppuration of the Brain, discovered by Dissection, but not suspected during Life.* (*P.* 89.)

A. Slough of the dura mater.

B. Abscess in the substance of the brain, with its upper surface turned back, and covered with white sloughs.

C. A probe passed out at the lower opening of the abscess, between the convolutions of the brain.

D. Cut edge of the falciform process of the dura mater.

E. Opening by which the abscess communicated with the lateral ventricle.

F. F. Whole extent of the ventricle laid open by a horizontal section.

G. Plexus choroides in a sloughy state.

H. Ulcerated surface of the thalamus nervi optici.

*Observations*

